# Barriers and facilitators to condom use among People Who Inject Drugs in Georgia: A qualitative study guided by the COM-B model

**DOI:** 10.1371/journal.pone.0346771

**Published:** 2026-04-13

**Authors:** Tamar Zurashvili, Maia Kajaia, Oluwabusayomi Akeju, Jack DeHovitz, Mamuka Djibuti

**Affiliations:** 1 Faculty of Medicine, Ivane Javakhishvili Tbilisi State University, Tbilisi, Georgia; 2 Partnership for Research and Action for Health, Tbilisi, Georgia; 3 University at Albany, State University of New York, Albany, New York, United States of America; 4 New York State Department of Health, Albany, New York, United States of America; 5 Department of Medicine, SUNY Downstate Health Sciences University, Brooklyn, New York, United States of America; United States Agency for International Development (USAID), NIGERIA

## Abstract

People who inject drugs (PWID) are at a heightened risk of contracting and transmitting HIV due to shared needles and unsafe sexual practices. Evidence on what shapes condom use among PWID in Georgia is limited. We conducted qualitative study among PWID recruited from two harm-reduction organizations in Tbilisi and Samegrelo between June-August 2025. Interviews were guided by the COM-B framework and analyzed thematically. Reduced sexual pleasure emerged as a dominant capability-related barrier to condom use. Knowledge of risks, benefits, and where to obtain condoms supported condom use, although participants noted that some community members lacked such knowledge. Opportunity factors were mixed. The convenient access to condoms, whether purchased at pharmacies or available for free at harm-reduction centers, created opportunity for condom access, and facilitated condom use; the factors impeding the use included the cost of purchase as well as long distance to and restricted hours of outlets, and occasional stock-outs and concerns about quality of condoms at harm-reduction centers. Social dynamics emerged as essential factors. Partner pressure, coercion, and persuasion, often intensified by alcohol or drug use, limited condom negotiation, whereas partner support, preparedness, and peer information sharing facilitated condom use. Motivation-related barriers included being under the influence of alcohol or stimulants and “heat of the moment” impulsivity, alongside low perceived risk with regular partners. Motivators included a strong desire for self and partner protection, heightened risk perception with casual or commercial partners, fear of infection, and past negative experiences including infections or unintended pregnancy. Findings indicate that condom use among PWID in Georgia is shaped by interplay of capability, opportunity, and motivation factors. Culturally sensitive, locally adapted strategies addressing pleasure-related concerns through education, strengthening negotiation and refusal skills, including couple-focused approaches can improve consistent condom use in Georgia and may benefit other countries in Eastern Europe and Central Asia.

## Introduction

People who inject drugs (PWID) continue to face dual risk of becoming infected with HIV occurring from engaging in unsafe injection practices such as needle or equipment sharing and other sexually transmitted infections (STIs) from participating in condomless sexual activity [1,2]. As reported by UNAIDS, PWID are approximately 14 times more likely to contract HIV than the general population [3]. Compared with non-injecting drug users, PWID experience overall poorer health outcomes and have heightened risks of acquiring bloodborne viruses such as hepatitis B and C, HIV and other STIs [4–9].

Globally, researchers estimated that 40.8 million people were living with HIV (PLWH) in 2024, with PWID representing about 7.1% of this population [10]. At the regional level, the numbers of new HIV infections continue to rise in the World Health Organization (WHO) Eastern European and Central Asia (EECA) region and consequently, the EECA region is not on track to meet the targets for ending AIDS by 2030. Evidence of HIV case reports in this region suggests that an increasing proportion of new infections arise from sexual transmission, and findings indicate that PWID, sex workers (SW) and men who have sex with men (MSM) are the main drivers of new infection [11,12]. Notably, reports indicate that the Republic of Georgia ranks high among the nations with the highest rates of PWID, with estimates from the Georgian AIDS and Clinical Immunology Research Center showing that nearly a third (30.1%) of the nation’s HIV cases occur among PWID [13].

Several studies have shown that PWID often engage in high levels of sexual risk behaviors, including having multiple sexual partners, participating in transactional sex and engaging in unprotected vaginal and anal sex, placing them at increased risk for HIV and STIs [14–17]. While injection related risk still exists, research has shown that sexual risk behaviors such as unprotected sex continue to be an important route of HIV transmission and other STIs among PWID [2,18]. Although, in the past, majority of HIV infections among PWID occurred from unsafe injection practices with a portion of cases resulting from unprotected sexual contact [19,20]. However, in more recent years, transmission through sexual activity has become the main driver of the epidemic and accounts for a growing increase in the number of new cases in Georgia. Significantly, in 2024, statistics showed that of the new HIV infection cases reported, heterosexual transmission accounted for 53.2%, while same sex transmission among MSM represented 13.7% [13].

To prevent the spread of STIs, correct and consistent condom use is universally recognized as one of the most effective methods of HIV and other STIs prevention [21]. Despite this efficacious means of protection, frequent condom utilization among PWID remains low and ranges from 11% to 51%, especially in developing counties [22]. Consistent condom use among PWID in Georgia is also suboptimal and findings from recent research conducted in Georgia showed that condom use was inconsistent, with less than 50% of PWID reporting regular use when involved with casual or commercial partners in the last 6–12 months [23–25]. This inconsistency in condom use among PWID has been linked to factors such as knowledge gaps, type of partner, condom accessibility and availability, decreased pleasure and low risk perception [22,26–28]. While studies have identified these determinants, they may vary across different populations and settings.

Hence, this study aimed to explore the barriers and facilitators to consistent condom use among PWID in Georgia with the goal of integrating sexual health components within the harm reduction services. The COM-B framework guided this research by examining how capability (physical, psychological), opportunity (physical, social) and motivation (automatic and reflective) influence condom use behavior among PWID in Georgia [29].

## Methods

### Setting

The study was conducted in collaboration with two organizations that are part of the Georgian Harm Reduction Network (GHRN). GHRN is a national umbrella association that brings together 26 member organizations across the country. Of these, 14 organizations specifically implement needle and syringe programs (NSPs) and provide a comprehensive package of harm reduction services, including sterile injecting equipment, condoms, HIV, HCV, HBV and syphilis testing, counseling, education, case management and linkage to health and social services. The remaining member organizations focus on other key populations and thematic areas, such as advocacy, community mobilization, and broader health and human rights initiatives.

For this study, two GHRN-affiliated organizations were selected – one located in Tbilisi, the capital of Georgia, and another in the Samegrelo region of western Georgia. These sites were chosen because both areas have among the highest HIV prevalence rates in the country. In addition, the Tbilisi site reflects an urban context, while the Samegrelo site represents a regional setting; including both locations allowed us to explore potential urban–regional differences in barriers and facilitators to condom use.

### Participants and recruitment

Participants were purposively selected with the assistance of social workers from two GHRN-affiliated organizations in Tbilisi and Samegrelo. The recruitment started on 25 June 2025 and continued until 20 August 2025. Inclusion criteria required that individuals of both sex be aged 18 years or older, have engaged in sexual activity within the past six months, have injected drugs within the past six months, and provide written informed consent. Individuals who did not meet these criteria or declined participation were excluded. Recruitment continued until the target sample size of ten participants was reached, with five participants recruited from Tbilisi and five from Samegrelo, while ensuring that thematic saturation was achieved.

### Data collection

A semi-structured interview guide, developed based on the COM-B framework, was used to explore barriers and facilitators to condom use among PWID (see S1 File for the interview guide). The guide was organized into sections addressing physical and psychological capability (e.g., knowledge, skills, and perceptions related to condom use), physical and social opportunity (e.g., availability, affordability, partner influence, and cultural norms), and automatic and reflective motivation (e.g., impulsivity, past experiences, risk perception, and protective intentions). Open-ended questions were followed by probes to elicit detailed narratives and examples. The guide was pilot tested with two participants to evaluate clarity, sequencing, and comprehensiveness; no substantive changes were required, and these pilot interviews were included in the analysis.

A total of 10 in-depth interviews were conducted remotely via Zoom. Each interview lasted 45–60 minutes. All interviews were conducted by one researcher with prior experience conducting qualitative research with vulnerable populations. At the start of each interview, the researcher emphasized confidentiality and voluntary participation and used a non-judgmental approach to encourage open discussion. Efforts were made to build rapport and create a comfortable environment so that participants could speak freely about sensitive topics. With participants’ written informed consent, all sessions were audio-recorded and transcribed verbatim to ensure accuracy and capture participants’ own wording. To compensate for their time, participants received 70 GEL (approximately USD 25).

### Data analysis

We used thematic analysis to examine patterns related to barriers and facilitators of condom use among PWID, following the six-phase approach described by Kiger and Varpio: (1) familiarization with the data, (2) generating initial codes, (3) searching for themes, (4) reviewing themes, (5) defining and naming themes, and (6) producing the report [30]. Verbatim transcripts were read repeatedly and annotated; two research team members independently manually coded an initial subset to develop a shared codebook, met to reconcile differences by discussion and refined the final codebook (see S2 File for the codebook). Codes and themes were organized deductively under the COM-B domains (Capability, Opportunity, Motivation), while allowing inductive subthemes to emerge within each domain.

Thematic saturation was reached during analysis, as no substantially new themes emerged in the later interviews. Although the total sample included ten participants, qualitative research suggests that thematic saturation in interview-based studies is often achieved with relatively small samples when the study scope is focused and participants share similar experiences [31]. In this study, participants were recruited from NSPs and shared similar experiences related to injection drug use, sexual behaviors and engagement with harm reduction services, which facilitated the identification of recurring themes. In addition, participants described behaviors they observed among peers in their communities, providing insights beyond their own experiences.

### Ethical considerations

Ethical approval for the study was obtained from the Institutional Review Board (IRB) of the Georgian National Centre for Disease Control and Public Health (# 2025−004). Potential participants were first approached by social workers from partner organizations, who explained the aims and objectives of the study and obtained written informed consent. Prior to each interview, the interviewer also obtained verbal consent for audio-recording. All research activities commenced only after IRB approval was secured.

## Results

**Fig 1:**
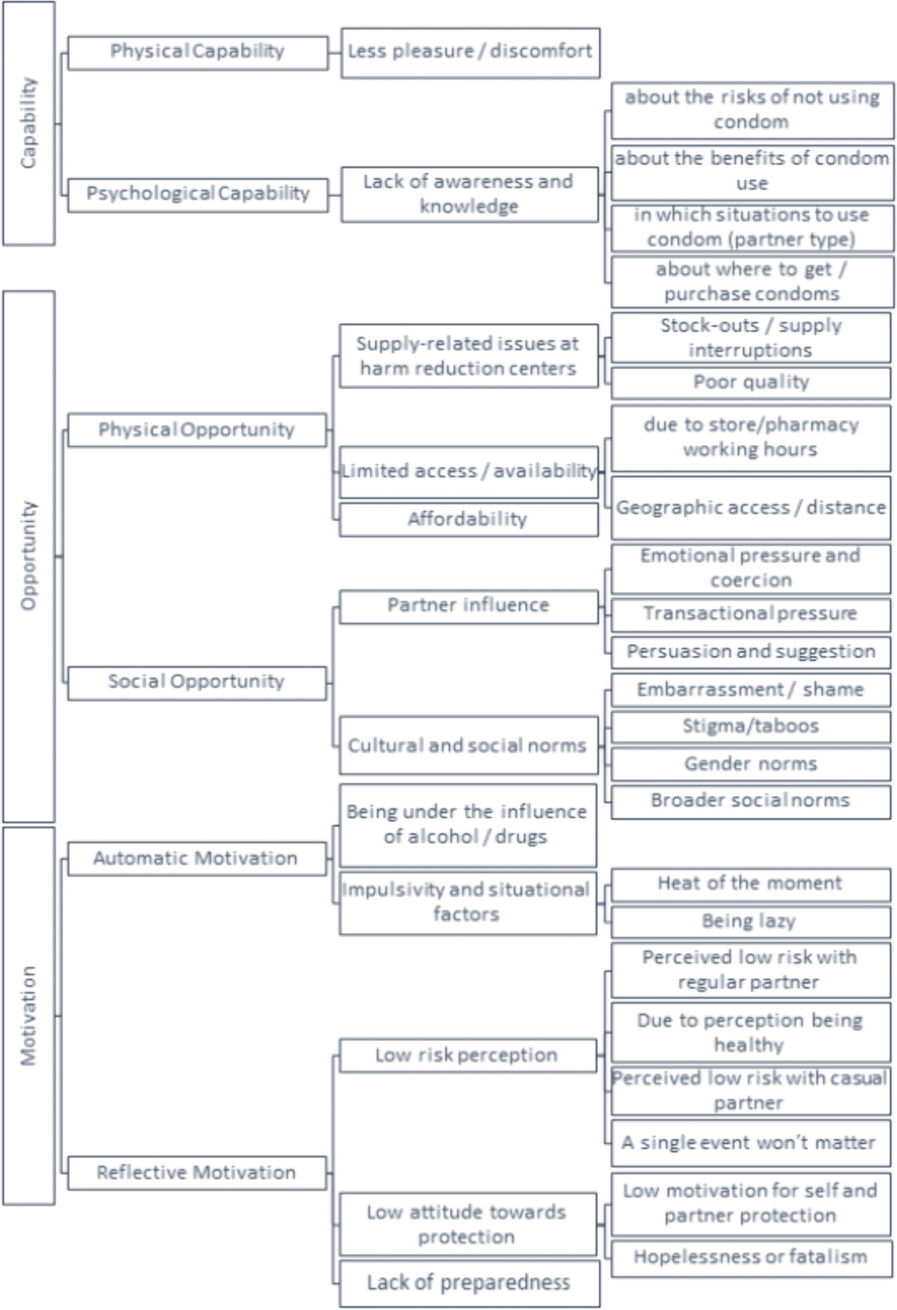
Barriers to condom use among PWID in Georgia.

**Fig 2:**
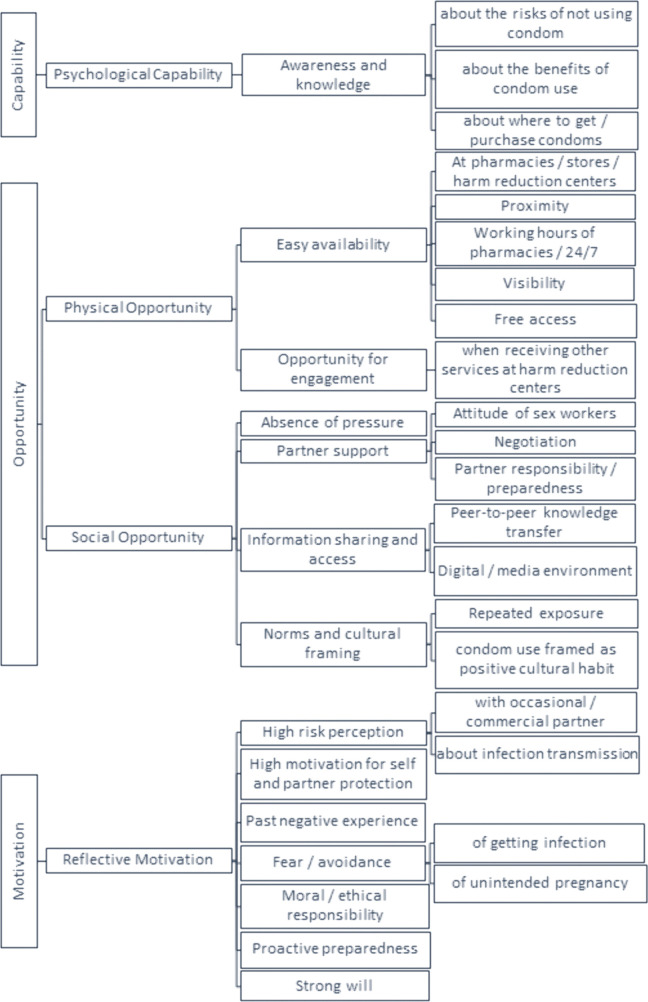
Facilitators to condom use among PWID in Georgia.

We organized findings using the COM-B framework across its three domains—Capability, Opportunity, and Motivation and coded themes as barriers or facilitators accordingly; the full coding hierarchy is depicted in Fig 1 (barriers) and Fig 2 (facilitators). To convey how commonly responses appeared across the 10 interviews, we used the following qualitative descriptors: “few” (1–2 responses), “some” (3–4), “most” (5–7), “nearly all” (8–9), and “all” (10). Overall, 10 PWID were interviewed between June and August 2025, including five from Tbilisi and five from Samegrelo. Participants’ ages ranged from 27 to 49 years, with a mean of 40.5 years (SD = 7.6) and a median of 42 years (IQR = 39.3–44.5), two females and 8 males.

### Capability

Reduced physical pleasure emerged as a significant Physical Capability barrier to condom use. All participants reported that either they themselves or their peers perceived sex with condom as less enjoyable. While most respondents emphasized reduced pleasure as a strong deterrent, one participant noted that the difference was relatively small in his own experience, and another suggested that the impact on pleasure is highly individual.

*“The reason is always the same — reduced pleasure. You know, “You can’t get a good grip with gloves on [you can’t get the same feeling with a condom]” and things like that. That’s just how it is.” –* ***45-year-old PWID, male, Tbilisi***

*“Yes, it’s also true that people feel reduced pleasure with a condom… many don’t use them for this reason… people sometimes say sex is better without [a condom] … but in my opinion, the difference is not that big. From my personal experience, I don’t see a significant difference…” –* ***49-year-old PWID, male, Tbilisi***

*“I’ve heard some people say they feel discomfort or can’t reach full pleasure with condoms… of course, sex without a condom feels different than with one… Still, yes, the pleasure without a condom is less… Among the people around me, there are some who say, “Why should I have sex like that if I can’t enjoy it?” So they don’t care about using condoms.” –* ***49-year-old PWID, male, Zugdidi***

Lack of awareness and knowledge was identified as a Psychological Capability barrier to condom use, primarily when participants described the experiences of other members of their PWID communities. Most emphasized that some peers might have limited understanding of the risks of unprotected sex, particularly underestimating the possibility of HIV, hepatitis, or other STIs transmission. Some also noted insufficient awareness of the benefits of condom use or a lack of clarity about when condoms should be used, for example with different partner types. Only one participant mentioned that some PWID might not be aware that condoms are distributed through harm reduction centers. In all these cases, lack of knowledge was perceived as a factor that could act as a barrier to condom use.

*“There are also those who don’t fully understand the risks of transmitting HIV, HCV, HBV, or other STIs… I’d say PWID know about the benefits roughly 50/50.” –* ***42-year-old PWID, female, Tbilisi***

*“I can’t say that I fully understand [the risks of not using a condom]… I think members of my community don’t understand these risks and don’t know about them — there’s a lack of knowledge in this regard… Accordingly, my community members also don’t know the benefits [of regular condom use]… Logically, you’d think they should have this knowledge, but from what I see around me and what we discuss, only a handful of people both use condoms and know the harm that comes from not using them, as well as the benefits of using them… There is lack of knowledge about in which situation to use condom as well, I mean the partner type.” –* ***28-year-old PWID, female, Tbilisi***

On the contrary, awareness and knowledge of the risks and benefits of consistent condom use, as well as where to obtain condoms, emerged as an important Psychological Capability facilitator to condom use among PWID. To a large extent, participants demonstrated good knowledge in these areas, though not always complete. Nearly all participants articulated awareness of the risks of unprotected sex, such as the transmission of HIV, hepatitis, and other STIs. Nearly all likewise emphasized the benefits of condom use, most often citing protection from infections and, in some cases, prevention of unintended pregnancy. All participants reported knowing where to access condoms, most commonly mentioning pharmacies and harm reduction centers as a source.

*“I think the majority are more aware of the risks… I know that there are many infections you can get from unprotected sex — it’s definitely possible.” –* ***42-year-old PWID, male, Tbilisi***

*“The risks are too serious — I know how things are transmitted, I’m fully aware, and I never have sex without a condom…” –* ***49-year-old PWID, male, Zugdidi***

*“Yes, I know that infections won’t be transmitted [when using condom]. Also, for example, if I’m healthy and my spouse is a user and I don’t want to have a child, I would use a condom and be protected…” –* ***49-year-old PWID, male, Tbilisi***

*“Yes, I think PWID know where to get condoms, mainly from pharmacies. We also know that they are distributed at harm reduction centers.” –* ***27-year-old PWID, male, Zugdidi***

### Opportunity

Limited access and availability was considered as a Physical Opportunity barrier to condom use in two directions. Some participants from the region talked about geographic barrier, such as distance to pharmacies or harm reduction centers, particularly when sexual encounters occurred in villages, forests, or other locations far from outlets**.** Some respondents both from the capital and the region, noted that pharmacy and store working hours might pose difficulties, as most of them are often closed at night when condoms are needed.

Some participants identified supply-related issues at harm reduction centers as a Physical Opportunity barrier, talking about experiences of stock-outs or supply interruptions and dissatisfaction with the quality of condoms provided.

Finally, few participants from the region identified affordability as a Physical Opportunity barrier, explaining that limited financial resources sometimes prevented them from purchasing condoms, particularly when they had multiple partners.

*“For a while, they [at harm reduction center] stopped providing them [condoms], so I was buying them, and then they brought them in again, although the quality was poor.” –* ***28-years-old PWID, female, Tbilisi***

*“Yes, a few years ago I had a situation where I wanted to use a condom but couldn’t get one — it was nighttime, the pharmacies were closed, and so I ended up having unprotected sex. I’ve also heard of similar cases from others, for the same reason.” –* ***40-years-old PWID, male, Zugdidi***

*“Yes, I’ve had 1–2 cases where I wanted to use a condom but couldn’t find one, because I was in a village where they weren’t available… If you have to buy condoms, it can mean spending a lot of money, especially if you have many partners... unfortunately, in Georgia, money for buying condoms is a problem. I don’t have spare cash for that either” –* ***49-years-old PWID, male, Zugdidi***

With regard to Physical Opportunity facilitators, easy access and availability of condoms was widely emphasized. All participants reported that condoms could be easily obtained at pharmacies or stores, and also available through harm reduction centers. Most highlighted the proximity of these outlets as an important enabler, while some participants specifically mentioned the advantage of 24/7 pharmacies. In addition, some participants referred to the visibility of condoms in pharmacies, and another three described the free access provided through harm reduction centers as a significant facilitator.

A further Physical Opportunity facilitator described by few participants was the opportunity for engagement created by harm reduction centers. They explained that visiting these centers not only allowed them to obtain condoms for free but also fostered regular interaction with services, which in turn reinforced consistent access and use.

*“Now [condoms] are very widely and easily available — in pharmacies, in stores. I also know they’re distributed at harm reduction centers… When I used to get syringes and other supplies from there [harm reduction center], they would give us condoms too. So, if I happened to be there, I would grab it, sure — but I wouldn’t go there specifically just for that.” –* ***45-years-old PWID, male, Tbilisi***

*“It makes me laugh to think they [PWID] couldn’t get one [condom]… They’re [condoms] sold everywhere — in stores, pharmacies… If you’re struggling and can’t afford to buy them, you can go there [harm reduction center] and they’ll give them to you…” –* ***49-years-old PWID, male, Tbilisi***

*“I live nearby, and it only takes me 10 minutes to get here [to the harm reduction center]. I also have a pharmacy close by… here [at harm reduction center] everything is free… Since I started coming here [at harm reduction center] and became a beneficiary, I come here for everything.” –* ***43-years-old PWID, male, Zugdidi***

*“I live in the city center, where 24-hour pharmacies and stores are available.” –* ***28-years-old PWID, female, Tbilisi***

Partner influence was frequently reported as a Social Opportunity barrier to condom use. Most participants described experiences of partner influence, most often in the form of emotional pressure and coercion, including accounts of being persuaded during intimate encounters and pressure escalating under the influence of alcohol. Some participants further referred to transactional pressure, where condomless sex was encouraged through offers of money or drugs. Others described influence in the form of persuasion and suggestion, such as partners presenting condomless sex as more pleasurable, expressing personal preference, or framing it as a mutual agreement.

Another Social Opportunity barrier identified was cultural and social norms, mentioned by some participants. They highlighted embarrassment or shame when buying condoms, particularly in smaller communities where participants feared being judged at pharmacies. One of them also pointed to stigma and taboos, describing old-fashioned mentalities and silence around sexual health, while another noted about the gender norms, citing the expectation that condom use was the man’s responsibility. Finally, broader cultural attitudes were described, such as the absence of a strong “culture of self-protection” within the community or presence of normative belief about having sex in a “real Georgian way”.

*“I’ve also heard of women being pressured — they [men] start with alcohol, then request sex without condom, then turn more aggressive… there are situations where, if there are drugs, someone might say, “I can spend time with you, we can get high together,” as a kind of offer.” –* ***42-years-old PWID, male, Tbilisi***

*“Yes, I had situation when I had condomless sex, and my partner justified it by saying that the feeling and pleasure are different without a condom. But he presented it all so nicely that I didn’t feel pressured — he persuaded me… In my opinion, it’s due to mentality — an old-fashioned mindset — they feel embarrassed to buy condoms or even to take them from harm reduction center… From a woman’s perspective, the perception and expectation are probably that it’s the man’s responsibility to put on a condom, and they shouldn’t have to remind him — so they often place that responsibility entirely on the man.” –* ***28-years-old PWID, female, Tbilisi***

*“They don’t have the culture of first protecting themselves and then thinking that if they have something, which they might not even know, they shouldn’t transmit it to others...* ***- 49-years-old PWID, male, Tbilisi***

Several Social Opportunity facilitators were identified, although most were mentioned by single participants. The absence of pressure was the most common theme, with nearly all participants stating that they had never been pressured by a partner not to use a condom, nor had they heard of such cases within their communities.

Another Social Opportunity facilitator was partner support for condom use, described in different ways. One participants emphasized that sex workers insist on condom use. Another described situations of successful negotiation, where women resisted male pressure and ultimately prevented condomless sex. In addition, few participants highlighted partner preparedness and responsibility, noting cases in which partners provided condoms or refused sex without them.

Information sharing and access also emerged as a Social Opportunity facilitator for condom use. One participant described peer-to-peer knowledge transfer, in which he actively reminded his peers to use condoms and seek services at harm reduction centers. Another referred to the digital and media environment, noting that platforms such as TikTok and the internet contribute to broader awareness of condom benefits.

Finally, few participant described norms and cultural framing as a Social Opportunity facilitator. One compared repeated exposure to messages about condoms to Coca-Cola advertisements, arguing that constant reminders reinforce the behavior. Another framed condom use as a positive cultural habit, comparing it to an everyday routine such as brushing one’s teeth.

*“Well, I’ve never had a situation like that [wanting to use condom but the partner refusing]. And I haven’t heard of it from others either… When you mentioned commercial partners — they themselves tell you that you have to use one [condom]. Otherwise, I know many people around me who, if they [sex workers] weren’t insisted on, wouldn’t use it [condom].”* ***– 45-years-old PWID, male, Tbilisi***

*“There were 1–2 times when I didn’t have a condom myself, but my partner did. I’ve been with people who protected themselves too — even if I had said I wanted sex without a condom, they wouldn’t have agreed.” –* ***49-years-old PWID, male, Zugdidi***

*“It’s the 21st century, and with the internet, TikTok, and everything else, you get this information [on benefits of condom use] from everywhere.”* ***– 40-years-old PWID, male, Zugdidi***

*“It’s like Coca-Cola — you know it does not need advertising, right? But we keep hearing those ads, and it’s a psychological thing — it gets into your head, and you always want it. It works the same way here: the more you hear about it [condom], the more you think about it.”* ***– 42-years-old PWID, male, Tbilisi***

### Motivation

Being under the influence of alcohol or drugs emerged as an important Automatic Motivation barrier to condom use. All participants described situations where being under the influence of alcohol or drugs reduced their ability to consider protective behaviors. Participants explained that intoxication often led to diminished attention to health, impaired memory, and impulsive decision-making, with many emphasizing that alcohol and drugs such as stimulants were particularly linked to risky sexual encounters.

In addition, some participants described impulsivity and situational factors as Automatic Motivation barriers. These included being caught up in the “heat of the moment”, sometimes tied to younger age and feeling too lazy to obtain a condom when one was not immediately available.

*“There are drugs that make you lose control — I’m not talking about opiates like heroin — but, for example, when people are on ecstasy, MDMA, or magic mushrooms, in such cases they might not use condoms…” –* ***42-year-old PWID, female, Tbilisi***

*“Of course, when you’re under the influence of drugs or alcohol, condom use is the last thing on your mind. Personally, I don’t think about it at all in those moments. Naturally, it’s hard to focus on your health at such times [when under the influence of drugs/alcohol].”* ***– 45-year-old PWID, male, Tbilisi***

*“As for others, not from drugs, but when drunk, yes — they’ve had sex without a condom, and later remembered it. They experienced burning sensations afterward and had to see a doctor. I’ve had one risky sexual encounter myself, when I was very drunk — I didn’t even think about using a condom, even though I had one in the car.”* ***– 49-year-old PWID, male, Zugdidi***

*“We were already so caught up in the moment that I definitely didn’t think about it [using condom].”* ***– 42-year-old PWID, male, Tbilisi***

*“Unfortunately, yes, I’ve had such an experience [wanted to use a condom but couldn’t get one]… I didn’t have a condom, and I was too lazy to go out and get one.”* ***– 42-year-old PWID, male, Tbilisi***

Low risk perception emerged as a significant Reflective Motivation barrier**.** All participants reported low risk perception with regular partners**,** often explaining that condom use was unnecessary with a spouse or trusted partner whom they believed to be “clean”. This pattern was consistently framed as a conscious decision based on trust and familiarity. Only one participant described a perceived low risk with a casual partner, framing it as a similar barrier to condom use. There were additional singular cases where participants justified condom non-use because they considered themselves healthy, or minimized risk by reasoning that a single unprotected encounter would not have consequences.

Another Reflective Motivation barrier to condom use concerned low attitude towards protection identified by most participants**.** This was expressed through apathy or lack of motivation to protect themselves or a partner, and in some cases through hopelessness or fatalism, described as “giving up” or believing “it does not matter what’s going to kill me.” Others emphasized a general disinterest in condom use, observing that many in their community did not value their health highly, failed to prioritize protective behaviors, or simply “did not care.”

Some participants from the capital described lack of preparedness as a Reflective Motivation barrier to condom use. They talked about situations where unprotected sex occurred in remote or spontaneous settings, such as in forests or recreational areas, where condoms were not readily available. However, these accounts reflected a lack of preparedness rather than structural inaccessibility, since condoms were otherwise widely accessible in urban areas. In these cases, the barrier arose from not planning ahead and failing to carry condoms.

*“With a regular partner, I know she’s mine and clean, so I might not use one... For example, if a couple goes up to Mtatsminda [central park in Tbilisi], they’ve been drinking and they get to that point [of having sex], and neither of them have a condom, they won’t be able to find one there. Naturally, they’ll end up having sex without it [condom]…” –* ***42-year-old PWID, male, Tbilisi***

*“It also depends on the partner. For example, I don’t use them with my regular partner… When you know who your partner is and she is your regular partner, then using a condom isn’t necessary… if it’s someone I trust and know well, I may not use a condom.”* ***– 43-year-old PWID, male, Zugdidi***

*“Motivation is probably individual. When you know you’re healthy, you see less risk and may be less likely to use condoms… As for condom use, many people in our community don’t even have the desire to live a healthier life… …I was like that too, and I think most of us drug users are the same — we’ve given up, with the attitude of “it does not matter what’s going to kill me. Yes, that’s exactly what I’m saying… they’ve given up… At exactly those times, it depends on the type of person — some may dismiss it and not use one [condom]. This group of people simply doesn’t care…”* ***– 49-year-old PWID, male, Tbilisi***

*“Some think, “It’s just this one time, nothing will happen to me,” there are such cases also.” –* ***42-year-old PWID, female, Tbilisi***

The most prominent theme within the Reflective Motivation facilitators was high motivation for self and partner protection, reported by nearly all participants. Respondents consistently emphasized their deliberate commitment to protecting both themselves and their partners, often describing condom use as a conscious, routine practice regardless of partner type. Notably, only one participant explicitly underlined the increased risk his injecting drug use could pose to a regular partner, which was a driver for partner protection.

Another strong Reflective Motivation facilitator was high risk perception, described by most participants in relation to occasional or commercial partners, and by some specifically regarding the risk of infection transmission. Participants explained that perceived risks were highest with non-regular partners, commercial sex encounters, or when infection was a possibility, and these circumstances consistently motivated condom use.

Past negative experiences also emerged as a Reflective Motivation facilitator, identified by most participants. These included histories of contracting hepatitis B or C, sexually transmitted infections, or other health complications, as well as experience of unintended pregnancy. These experiences were described as important turning points that encouraged consistent condom use.

Further Reflective Motivation facilitator to condom use was fear or avoidance of negative outcomes. Most participants described fear of acquiring HIV or other infections, while some participants emphasized avoiding unintended pregnancies as an important reason for condom use.

Finally, some participants mentioned other Reflective Motivation facilitators. Two of them referred to a moral or ethical responsibility to protect partners, particularly when aware of their own infection status. Singular accounts described strong will and proactive preparedness as additional facilitators to consistent condom use.

*“But otherwise [apart from regular partner], in 99% of cases, it’s risky. With commercial partners, the risks are even higher… with casual partners or commercial partners, you must use one [condom] 100%...”-* ***49-year-old PWID, male, Zugdidi***

*“I can’t and won’t approach a woman without a condom. After all, I’m a drug user, and something might be wrong with me without me even knowing it — and I don’t want to transmit it to my partner.”* ***– 49-year-old PWID, male, Tbilisi***

*“I once had a case where I caught a “bouquet” [a slang term for multiple infections]. I thought “bouquet” only meant flowers, but when I went to the doctor, I found out I had a lot of different things. Since then, I always use a condom with casual partners…”* ***– 42-year-old PWID, male, Tbilisi***

*“Both the fear of infection and of unwanted pregnancy are equally important motivators for me… Ideally, motivation shouldn’t depend on the type of partner, protection should be your responsibility, but in reality it does.”* ***– 27-year-old PWID, male, Zugdidi***

## Discussion

This study provides one of the first in-depth examinations of the barriers and facilitators to condom use among PWID in Georgia, using the COM-B framework to structure analysis. Several key themes emerged across the domains of Capability, Opportunity, and Motivation. Reduced sexual pleasure dominated as a Physical Capability barrier, while knowledge of risks and benefits functioned as a key Psychological Capability facilitator. However, participants noted that some community members still lacked such knowledge. Social dynamics played a central role with partner influence being the most powerful barrier and partner support an important facilitator. Within Motivation, condom use was hindered by alcohol and drug use, low perceived risk with regular partners, and apathy, but facilitated by strong motivation for self and partner protection, heightened risk perception with casual partners, fear of infection, and past negative experiences.

In this study, reduced physical pleasure was identified as the primary Physical Capability barrier to condom use, consistent with global research showing that perceptions of diminished sensitivity and altered sexual experience are among the most common reasons for condom non-use [32–34]. Evidence from the EECA region is limited, but the available study similarly indicated that reduced pleasure remains a salient barrier [35].

Knowledge and awareness about the risks of unprotected sex, the benefits of protected sex, and where condoms can be obtained emerged as the strongest Psychological Capability facilitators in our sample. This pattern accords with some global and regional evidence suggesting that greater HIV and sexual risk knowledge and awareness are linked to safer sexual practices among PWID [36,37]. At the same time, the lack of knowledge and awareness about the risks of unprotected sex, the benefits of protected sex, and where condoms can be obtained also functioned as a barrier when participants described peers who lacked such knowledge. These gaps have been shown to contribute to condom non-use [38–40].

Easy access and availability of condoms was identified as a key Physical Opportunity facilitator in our study. Condoms were described as widely obtainable at pharmacies, stores, and harm reduction centers, with additional enabling factors such as 24/7 outlets, visible placement, and free distribution through NSP services. These findings highlight that when condoms are consistently available in multiple, convenient locations, uptake is facilitated. Similar evidence from other settings shows that expanding the distribution of condoms, particularly through community and pharmacy channels, increases both access and use [41]. Importantly, participants also described harm reduction centers not just as supply points but as places where routine visits for basic services (e.g., needle and syringe exchange) created opportunities to access condoms and maintain regular contact with services, thereby reinforcing consistent condom access and use. This highlights the importance of service integration and echoes evidence that combining condom distribution with broader harm reduction and HIV prevention programs improves both access to supplies and ongoing engagement with services, leading to more consistent protective practices and person-centered care [42,43].

Within the COM-B domain of Physical Opportunity, participants described several barriers to consistent condom use. These included geographic constraints in regional areas, including restricted pharmacy hours, occasional stock-outs and poor-quality supplies at harm reduction centers, and, for some, the affordability of condoms. Collectively, these findings emphasize how structural and service-level limitations can undermine timely and reliable access. Similar challenges have been documented in other contexts, where distance to outlets, limited operating hours, and supply interruptions reduced condom uptake, while even modest costs deterred use among persons at risk for HIV and other STIs [35,44–46].

In this study, partner influence emerged as the strongest Social Opportunity barrier to condom use, with most participants reporting experiences of emotional pressure, coercion, or persuasion during sexual encounters. These barriers could be partly rooted in shared factors such as reduced sexual pleasure and limited knowledge. Partner influence was often intensified in the context of alcohol use, in line with the evidence from other settings where partner pressure substantially reduced condom negotiation power, particularly for women and those in vulnerable positions [47]. Beyond coercion, some participants described transactional contexts, where offers of drugs or money were used to encourage condomless sex. This parallels the findings from studies among PWID and sex workers that highlight the intersection of economic vulnerability and reduced condom use [48–50]. Some participants described less overt forms of persuasion, where partners framed condomless sex as more pleasurable or justified it as a mutual agreement. Such relational negotiations highlight that condom decision-making is rarely an individual choice but often shaped by interpersonal dynamics [51,52]. In addition to partner influence, some participants pointed to cultural and social norms as Social Opportunity barriers to condom use, though these were less frequently reported. Embarrassment or shame when purchasing condoms, gendered expectations that placed responsibility for condom use on men, and broader taboos around sexual health were highlighted. While these accounts were not dominant in our sample, they reflect well-documented barriers in other global and regional research showing that stigma and embarrassment around condom acquisition, gendered expectations in relationships, and norms that undervalue STI prevention collectively undermine condom use [28,36,53–56]. Taken together, these findings show that condom use is shaped not only by individual factors but also by interpersonal dynamics and cultural norms, which should be integrated into culturally sensitive prevention strategies for PWID.

In contrast to the barriers described above, several Social Opportunity facilitators emerged, though most were mentioned by only a few participants. The most common was the absence of partner pressure, essentially the opposite of the coercion and persuasion highlighted as barriers. Similarly, partner support, proactive preparedness, and sense of responsibility showed positive dynamics that contrasted with situations where partners refused or discouraged condom use. Peer information sharing and broader digital and media exposure also reflect how social environments can normalize protective behavior, a dynamic that has been documented in other studies [28,57]. Overall, these findings suggest that the same interpersonal and cultural domains that can act as barriers also hold potential to reinforce safer sexual practices when mobilized positively.

In our study, being under the influence of alcohol or drugs, particularly stimulants clearly emerged as a dominant Automatic Motivation barrier to condom use. Beyond substance effects, some participants also described “heat of the moment” or impulsive decision-making during intimate encounters as a barrier to condom use within the same COM-B domain. This aligns with prior global and regional studies among HIV key populations showing that substance use is strongly associated with condomless sex [58–62]. From a neurocognitive perspective, alcohol and stimulant use are known to heighten arousal and impulsivity while impairing executive control, whereby attention narrows to immediate cues and long-term risks are overlooked [63,64]. These processes align with the COM-B model’s concept of Automatic Motivation, in which behavior is shaped by urges and emotional states rather than deliberate reflection [29].

A central Reflective Motivation barrier in this study was low perceived risk with regular partners, consistently described as a conscious decision not to use condoms with trusted or long-term partners. Similar patterns have been documented globally, where individuals in stable relationships often discontinue condom use due to assumptions of mutual monogamy or a belief that their partner is “safe,” despite persistent HIV and STI risks among key populations or HIV-discordant relationships [65–67]. Other explanations for condom non-use, though less commonly reported in our study, can also be understood within this same logic of perceived low risk. Some participants justified condomless sex by reasoning that they were healthy or that a single encounter would not matter, reflecting a broader tendency to underestimate personal vulnerability. Low attitudes towards protection, reflected in apathy, hopelessness, and fatalism, emerged as another Reflective Motivation barrier to condom use. Similar findings have been reported in other studies, where apathy among drug users is linked to lower prioritization of health and reduced confidence in protective behaviors [68,69]. Within COM-B, this highlights how weakened motivation can discourage condom use but also points to opportunities for interventions that strengthen motivation.

High motivation for self and partner protection emerged as the strongest Reflective Motivation facilitator, with nearly all participants describing condom use as a deliberate and routine choice. This protective approach has been similarly highlighted in other studies where heightened awareness of partner risk and personal responsibility promoted consistent condom use among PWID and other key populations [70,71]. While it stands in contrast to the low attitudes towards protection as discussed above, it highlights how opposite motivational orientations can either strengthen or undermine protective behaviors. High risk perception with occasional or commercial partner functioned as another Reflective Motivation facilitator in this study, operating as the converse of low perceived risk with regular partners. This distinction underscores how risk appraisal was highly partner-specific: while trust and familiarity discouraged condom use in steady relationships, perceiving occasional or commercial partners as higher risk consistently motivated protection. Similar patterns were reported in a systematic review, where condom use was higher with casual and commercial partners than with regular ones [72]. At the same time, fear of infection, another Reflective Motivation facilitator in our sample, could be viewed as closely tied to high risk perception of transmission, with some participants describing avoidance of HIV or other STIs as a key motivator. Similarly, past negative experiences with infections or unintended pregnancies acted as Reflective Motivation facilitators, reinforcing these perceptions of high risk and further strengthening motivation for condom use. Similar findings have been reported globally among diverse populations [73–75].

Overall, these findings underscore that condom use among PWID in Georgia is influenced not only by individual capability and motivation but also by social and structural dynamics. Recognizing that barriers and facilitators often mirror each other within the COM-B domains highlights concrete entry points for intervention. Tailored strategies could include integrating brief counseling on condom use and condom negotiation skills into existing harm reduction services. Interventions could also address concerns about reduced pleasure by providing information on different types of condoms and lubricants. In addition, harm reduction service providers could reinforce protective motivations by discussing partner risk and shared responsibility during routine visits. Given the central role of interpersonal dynamics, couple-based interventions [76–78] that foster joint decision-making and shared responsibility for protection may be especially valuable. Primarily, interventions must be adapted to local context, taking into account regional realities, cultural norms, and the lived experiences of PWID. Training harm reduction staff to deliver such interventions and engaging peer educators or community members in awareness activities could further strengthen the acceptability and reach of such interventions. By grounding prevention strategies in these nuanced insights, programs can move beyond generic messaging to deliver person-centered, contextually relevant support that strengthens condom use and reduces HIV and STI transmission.

This study has several limitations that should be acknowledged. First, given the sensitive nature of sexual behavior and condom use, responses may have been influenced by social desirability bias, with participants potentially underreporting risk behaviors or overstating protective practices. Second, the study involved a relatively small sample size; however, qualitative data collection continued until thematic saturation was achieved. Importantly, participants not only shared their own experiences but also talked about the practices of peers in their communities, which provided broader insights beyond individual accounts. Third, there was the gender imbalance among participants, with only two women included in the sample. This may limit the extent to which the findings capture gender-specific barriers and facilitators to condom use among women who inject drugs. The distribution reflects the gender composition of clients attending needle and syringe programs in the study locations, where women are generally underrepresented. Future research should specifically explore the experiences and needs of women who inject drugs to better understand gender-specific factors influencing sexual risk behaviors. Another limitation of this study is that it did not explore broader structural factors that may influence sexual risk behaviors among PWID, such as poverty, policing practices, or stigma in healthcare settings. Future research should examine these broader contextual influences to better inform risk-reduction interventions. Finally, this study was conducted within the specific social, cultural, and health system context of Georgia. Therefore, some findings may not be directly generalizable to other settings where harm reduction services, social norms, and policy environments differ. However, the results may still be relevant for similar contexts in the EECA region, where comparable factors influence HIV risk among PWID. Despite these limitations, this study offers valuable evidence on the multi-level factors influencing condom use among PWID in Georgia. By applying the COM-B model, it contributes context-specific knowledge that can inform the design of tailored, culturally sensitive interventions and adaptations within harm reduction services to strengthen HIV prevention efforts in the region.

## Conclusion

This study is among the first to apply the COM-B framework to explore condom use barriers and facilitators among PWID in Georgia. We show that condom use behavior is shaped by three areas working together: capability, which includes what people know and how condoms are experienced; opportunity, which includes ease of obtaining condoms and the influence of partners and social norms; and motivation, which includes how people perceive risk and how alcohol or drugs affect decisions. To improve consistent condom use among PWID programs should address pleasure-related concerns through education; strengthen negotiation and refusal skills and partner responsibility; and include counseling that links substance use to sexual risk. Adapting these steps to local context and delivering them through person-centered services can help reduce HIV and STI transmission among PWID in Georgia. As these challenges are common across EECA, this approach is likely to benefit countries throughout the region.

## Supporting information

S1 File In-depth interview guide. (OCX)

S2 File Codebook. (DOCX)
