# Supplementary material for: Barriers and facilitators to condom use among People Who Inject Drugs in Georgia: A qualitative study guided by the COM-B model
Source: PLoS One. 2026 Apr 13;21(4):e0346771. doi: 10.1371/journal.pone.0346771 (PMC13075659; doi:10.1371/journal.pone.0346771)
Supplement: S1 File — (OCX) [file pone.0346771.s001.docx]

# COM-B-Based In-Depth Interview Guide for PWID

## Section 1: Capability

### 1.1 ****Physical Capability****

Condom use barriers

1. In your opinion, what are the main barriers to condom use among PWID in Georgia? You can talk about your own experience or experience that you’ve heard from your community members?
   - Probe: Do you think PWID in Georgia feel physically comfortable using condoms? discomfort during sex, physical pleasure, performance, etc.? What about your own experience?
   - Probe: What about the partner type and condom use barriers?

### 1.2 Psychological Capability

Knowledge, understanding, and decision-making related to condom use.

1. Do you feel you understand the risks of not using condoms with casual or paid partners? What about other members of your community?
   - Probe: HIV, HCV, HBV, another STI?
2. What do you know about the health benefits of consistent condom use? What about other members of your community?
   - Probe: How do you think does consistent condom use protect you from HIV or other STIs?
3. In which situations should PWID use condom?
   - Probe: Partners type? Any other?

## Section 2: Opportunity

### ****2.1 Physical Opportunity****

*Access to condoms and supportive environments for condom use*

1. Do you think PWID in Georgia know where to get/purchase condoms? Tell me about yourself as well
2. How easy is it to get condoms where you live or spend time?
   - Probe: clinics, pharmacies, or harm reduction centers, other in your area?
3. Are there times when you wanted to use a condom but couldn’t get one?
   - Probe: What usually prevents you from getting one—distance (Tbilisi/rural), cost, store hours, or something else?
   - Probe: Have there been situations where lack of access led to unprotected sex?

### ****2.2 Social Opportunity****

*Norms, social support, and attitudes that influence behavior*

1. Have you ever been pressured not to use a condom? Have you heard of such experience among your community members?
   - Probe: Was the pressure emotional, physical, or related to money or drugs?
   - Probe: Who made you feel pressured in that situation?
   - Probe: How did you respond in those situations, and how did it make you feel afterward?

## Section 3: Motivation

### ****3.1 Automatic Motivation****

*Emotions, impulses, and habits that influence condom use*

1. Are there situations when you don’t really think about it and just skip using a condom? Have you heard of such case among your community members?
   - Probe: Do you think this happens more often when you're (PWID are) under the influence of drugs or alcohol? Anything else?
   - Probe: Do you think your drug use makes it harder to stay focused on protecting yourself?

### ****3.2 Reflective Motivation****

*Intentions, beliefs, and conscious decision-making related to condom use*

1. How motivated are you to protect yourself and your partners from HIV and STIs?
   - Probe: What influences your motivation—fear of infection, relationship trust, past testing experiences?
   - Probe: Do you think your motivation changes depending on the partner or situation?
