# Supplementary material for: Barriers and facilitators to condom use among People Who Inject Drugs in Georgia: A qualitative study guided by the COM-B model
Source: PLoS One. 2026 Apr 13;21(4):e0346771. doi: 10.1371/journal.pone.0346771 (PMC13075659; doi:10.1371/journal.pone.0346771)
Supplement: S2 File — (DOCX) [file pone.0346771.s002.docx]

**Codebook**

| **COM –B** | **COM –B construct** | **Barriers**  **/Facilitator** | **Code** | **Child code** |
| --- | --- | --- | --- | --- |
| **Capability** | **Physical Capability** | Barrier | Less pleasure / discomfort | - |
|  | **Psychological Capability** | Barrier | Lack of awareness / knowledge / information | About risks of not using condom |
|  |  |  |  | The benefits of condom use |
|  |  |  |  | In which situations to use condom (partner type) |
|  |  |  |  | Where to get/purchase condoms |
|  |  | Facilitator | Awareness /knowledge | About the risks |
|  |  |  |  | About the benefits of consistent condom use |
|  |  |  |  | Where to get/purchase condoms |
| **Opportunity** | **Physical Opportunity** | Barrier | Supply-related issues at harm reduction centers | Stock outs / supply interruptions  (at harm reduction center) |
|  |  |  |  | Poor quality  (at harm reduction center) |
|  |  |  | Limited access / availability | Due to store/pharmacy working hours. |
|  |  |  |  | Geographic access/ distance |
|  |  |  |  | Not available at multiple places |
|  |  |  | Affordability |  |
|  |  | Facilitator | Easy availability | In pharmacies/stores  At harm reduction centers |
|  |  |  |  | Proximity |
|  |  |  |  | Working hours of pharmacies / 24/7 |
|  |  |  |  | Visibility |
|  |  |  |  | Free access |
|  |  |  | Opportunity for engagement | Access to condoms when receiving other services at NSPs. |
|  | **Social Opportunity** | Barrier | Partner influence | Emotional pressure and coercion |
|  |  |  |  | Transactional pressure (offers of money, offers of drugs) |
|  |  |  |  | Persuasion and suggestion |
|  |  |  | Cultural and social norms | Embarrassment /shame |
|  |  |  |  | Stigma/taboos |
|  |  |  |  | Gender norms |
|  |  |  |  | Broader social norms |
|  |  | Facilitator | Absence of pressure |  |
|  |  |  | Partner support for condom use | Attitude of sex workers |
|  |  |  |  | Negotiation |
|  |  |  |  | Partner responsibility / preparedness |
|  |  |  | Information sharing and access | Peer-to-peer knowledge transfer |
|  |  |  |  | Availability of information online / digital/media environment as information source |
|  |  |  | Norms and cultural framing | Repeated exposure |
|  |  |  |  | Condom use framed as a positive cultural habit |
| **Motivation** | **Automatic Motivation** | Barrier | Being under the influence of drugs |  |
|  |  |  | Impulsivity and situational factors | Heat of the moment (sometimes tied to younger age |
|  |  |  |  | Being lazy |
|  | **Reflective Motivation** | Barrier | Low risk perception | Perceived low risk with regular partner |
|  |  |  |  | Perceived low risk with casual |
|  |  |  |  | Due to perception being healthy |
|  |  |  |  | Perceived low risk of infection (A single event won’t matter) |
|  |  |  | Low protective orientation | Low motivation for self and partner protection |
|  |  |  |  | **Hopelessness or fatalism** |
|  |  | Facilitator | High risk perception | With occasional/commercial partner |
|  |  |  |  | About infection transmission |
|  |  |  | High motivation for self and partner protection |  |
|  |  |  | Past negative experience |  |
|  |  |  | Fear / avoidance | Of getting infection |
|  |  |  |  | Of unintended pregnancy |
|  |  |  | Strong will |  |
|  |  |  | Proactive preparedness |  |
|  |  |  | Moral/ethical responsibility |  |
